# Accident and emergency department attendance rates of people experiencing homelessness by GP registration: a retrospective analysis

**DOI:** 10.3399/bjgpopen20X101089

**Published:** 2020-11-04

**Authors:** Johanna Reilly, Khalil Hassanally, John Budd, Stewart Mercer

**Affiliations:** 1 Academic Clinical Fellow, Usher Institute, University of Edinburgh, Edinburgh, UK; 2 GP, Edinburgh Access Practice, NHS Lothian, Edinburgh, UK; 3 Professor of Primary Care, Usher Institute, University of Edinburgh, Edinburgh, UK

**Keywords:** inequalities, community care, socioeconomic factors, emergency service, hospital, harm reduction, people experiencing homelessness

## Abstract

**Background:**

People experiencing homelessness are known to have complex health needs and to be high users of hospital accident and emergency (A&E) departments. It is unclear whether access to a day-time specialist homeless medical practice, as opposed to routine general practice, influences A&E attendance rates.

**Aim:**

This study investigated whether registration with a specialist homeless service would alter A&E attendance rates in a single geographical region in Scotland.

**Design & setting:**

A health board area with a specialist service for people experiencing homelessness was selected. Data were obtained from the hospital records of 4408 A&E attendances by people experiencing homelessness at NHS Lothian (based on a broad definition of homelessness and including those in temporary accommodation) between January 2015 and July 2017.

**Method:**

The attendances were compared between people registered with a specialist service and those registered with a mainstream GP.

**Results:**

The reasons for attendance and urgency of attendance were broadly similar between the two groups. Repeat attendance was similarly high in both groups. Almost 70% in both groups attended with problems deemed urgent, very urgent, or requiring immediate resuscitation. The patients registered with the specialist homeless service were more likely to be older and male; however, this did not affect the frequency of attendance.

**Conclusion:**

People experiencing homelessness attending A&E mainly do so for urgent or very urgent problems. This was not related to the type of day-time primary care service they had access to. Strategies to reduce attendances, such as out-of-hours mobile medical units, should be explored.

## How this fits in?

People experiencing homelessness are known to have high attendance to A&E departments. It has been suggested poor access to primary care services may be a cause for this increased attendance, but it was found that access to primary care services did not affect attendance rate in this group. As most attendances were urgent and frequently related to drugs and alcohol, it may be that harm reduction strategies are needed to reduce the frequency of emergency attendance and resulting morbidity.

## Introduction

Homelessness is a complex health and social problem with multiple downstream determinants ranging between individual factors, such as substance misuse and mental health problems, and structural inequalities, such as lack of availability of housing and the welfare system.

Rates of homelessness have increased in the past 10 years in most high-income countries.^1^ In England rough sleeping has increased by 169% from 2009 to 2016, and local authority homeless applications have increased by 30%.^2^

People who are experiencing homelessness have worse physical and mental health than the general population, and are more likely to attend A&E. The association of homelessness with multimorbidity has been documented in several settings.^3,4^ People experiencing homelessness are particularly likely to have physical ill-health combined with mental illness and drug or alcohol abuse. Studies from the US and Canada have also found an earlier onset of frailty in people experiencing homelessness.^5^ Several studies have documented a high rate of A&E use in people experiencing homlessness. A 2016 study of people registered with a homeless health service found they had a rate of A&E attendance around 60 times that of the general population.^6^ In addition a larger study in Ireland comparing the A&E use of people experiencing homelessness and that of housed individuals found that compared with housed individuals, people experiencing homelessness had a 20 times increased use of A&E, and approximately 90% of the homeless population in the study presented to A&E during the 1-year study period.^7^ People experiencing homelessness may also account for an increased proportion of those termed 'frequent attenders' (defined as >6 attendances in 6 months). A study of A&E frequent attenders in England in 2000 found people experiencing homelessness made up 7.5% of all frequent attenders.^8^

Despite people experiencing homelessness clearly being in need of medical care, providing care can be difficult owing to often chaotic social circumstances and other barriers, such as a lack of money for transport to appointments and difficulty communicating with patients with no fixed address. Provision of appropriate treatment has the potential to save lives; for example, a recent English study linking death records and homeless services found a high rate of premature mortality among people experiencing homelessness, and that one in three such deaths were classed as amenable to medical care.^9^

It has been suggested that the high rate of A&E attendance by people experiencing homelessness could be because of difficulty in accessing appropriate primary care services.^8^ People experiencing homelessness can face multiple barriers to accessing GP care;^10,11^ for example, some surgeries may ask for documentation, which they do not have; appointment systems can be difficult to negotiate for people with additional needs or substance abuse who may have no one to advocate for them; and they may face prejudice from the public or staff.^12^

In response to this problem, there are various forms of specialist provision of primary care for people experiencing homelessness in the UK. These follow a variety of models; some provide only a limited service and do not fully register patients, whereas others attempt to provide a full and holistic service with mental health and drug and alcohol misuse treatment, as well as standard GP care and outreach to the homeless community.

A 2016 study of homeless primary care centres in England recognised there were multiple models of specialist health provision for people experiencing homelessness. This provision ranged from specialist health centres providing full GP registration and with an associated multidisciplinary team from one fixed site, to mobile and volunteer services providing a more limited range of healthcare services and not usually registering patients as the regular GP; for example, in this case the patient would remain registered with another GP.^13^ The same study found homeless health provision across England to be patchy, with some areas having no specialist service for people experiencing homelessness and few providing a fully comprehensive primary care service for this patient group.

In Scotland homelessness is a significant problem, with 8% of the Scottish population experiencing it at some point during their lifetime.^14^ The exact numbers of people experiencing homelessness at any one time in any particular geographical area is difficult to define; however, in Lothian, where this study took place, in 2017–2018 there were 3119 homeless applications made to the local council.^15^

In Edinburgh there is a GP practice providing care exclusively to people experiencing homelessness or to people insecurely housed, which is referred to as a specialist homeless health provider (SHHP). It is a GP practice based from two fixed sites in Edinburgh city. The SHHP aims to provide comprehensive care to patients; for example, the service has three part-time GPs, a mental health team, drug and alcohol treatment is provided and treatment for hepatitis C, there is harm reduction advice, dentistry, occupational therapy and podiatry, as well as third-sector involvement and outreach work. The practice has around 900 registered patients at any one time and appointments are available on weekdays both on a walk-in and booked basis. Out-of-hours cover is provided by NHS 24 and Lothian unscheduled care as in all GP practices in the region.

In the present study, A&E attendances were retrospectively compared over a 2.5-year period of people who were experiencing homelessness at that time to see if registering with a SHHP would alter the pattern of attendance compared with those registered with a mainstream GP. Although mainstream general practices can also provide care for drug and alcohol addiction and mental health, this would often initially be through referral and usually all services would not be available at the same site. In addition, mainstream GP appointments systems vary and, in general, would be less geared to the needs of people experiencing homelessness who may find attending appointments at a set time challenging.

## Method

A&E attendances at NHS Lothian from January 2015 to July 2017 were retrieved. The full record of each consultation was accessed. Permission for the data download was granted by the Caldicott Guardian.

Patients experiencing homelessness were filtered by a combination of factors:

- Patient registered at the SHHP.
- Patient registered on the system as 'no fixed abode' (NFA).
- Free-text search was carried out for the term 'homeless', 'no fixed abode', and 'NFA', and if the consultation history suggested that they were experiencing homelessness then the patient was selected.
- A list of hostels and temporary accommodation from the local authority was gathered, addresses were cross-referenced against this information. If a patient’s address was on the list they were included in the study.

The text of the consultations was analysed and the reason for attendance, whether drugs and alcohol was a consideration were extracted, and data were collated into an excel spreadsheet.

The dataset is likely to be an underestimate of the true number of people experiencing homelessness attending A&E as those who were 'sofa surfing' and who may have given a friend's or relative's address would not have been included, but this represents the best attempt to capture the homeless population attending for A&E care. Patients attending GP out-of-hours or contacting NHS 24 were not included. Rough sleeping was defined as patients with 'NFA' or 'not known' in the address field on presentation to A&E.

### Data analysis

IBM SPSS Statistics (version 24) was used for all statistical analysis.

Attendances were grouped into SHHP patients and non-SHHP patients, and differences between groups and frequencies of attendance were compared. A small proportion of attendances had no known GP or a registered GP outside Scotland and these were excluded from the analysis. The GP registered for the patients first attendance to A&E was used in the analysis.

Statistical significance of difference between groups was calculated using the Mann-Whitney U test for independent group. Differences in mean frequencies of attendance was calculated using the independent samples *t*-test.

## Results

A total of 4408 attendances were identified from 1225 people experiencing homelessness who attended A&E from January 2015 to July 2017. Of these, 304 (7.0%) attendances were excluded as the GP was unknown. Of the remaining 4099, 2545 (62.1%) were from the SHHP and 11 559 (37.9%) from other GP practices.

This represents only a small proportion of total A&E attendances in this health board area, which had an A&E attendance rate in 2015 of approximately 204 000 attendances.^16^

SHHP patients were on average significantly older and were more likely to be male than other GP patients. There was no statistically significant difference in rates of rough sleeping between groups (Table 1).

**Table 1.** Demographics and reasons for attendance

**Sex^a^**	**SHHP, *n* =** **670, *n* (%)**	**Other, *n* =** **454**, *n* (%)****	**Significance**
Male	524 (78.2)	275 (60.6)	*P* = 0.001
Female	146 (21.8)	178 (39.2)	
**Age range, years^a^**	**SHHP, *n* =** **670**	**Other, *n* =** **454**	*P* = 0.001
18–24	58 (8.7)	93 (20.5)	
25–29	97 (14.5)	76 (16.7)	
30–34	142 (21.2)	79 (17.4)	
35–39	110 (16.4)	81 (17.8)	
40–44	103 (15.4)	47 (10.4)	
45–49	44 (6.6)	17 (3.7)	
50–54	30 (4.5)	16 (3.5)	
55–59	15 (2.2)	8 (1.8)	
60–64	6 (0.9)	3 (0.7)	
65–69	6 (0.9)	3 (0.7)	
≥70	3 (0.4)	5 (1.1)	
**Rough sleeping^a^**	**SHHP, *n* =** **670**	**Other, *n* =** **454**	*P* = 0.839
Yes	112 (16.7)	78 (17.2)	
No	558 (83.3)	376 (82.8)	
**Reasons**	**SHHP, *n* =** **2545**	**Other, *n* =** **1554**	*P* = 0.026
Medical	540 (21.2)	353 (22.6)	
Surgical	150 (5.9)	134 (8.6)	
Trauma	376 (14.8)	272 (17.4)	
Alcohol	385 (15.1)	182 (11.7)	
Drugs	579 (22.8)	197 (12.6)	
Mental health	164 (6.4)	189 (12.1)	
Unknown or did not wait	229 (9.0)	113 (7.2)	
Social or other	122 (4.8)	119 (7.6)	
**Triage**	**SHHP, *n* =** **2545**	**Other, *n* =** **1** **554**	*P* = 0.08
Immediate resus	165 (6.5)	75 (4.8)	
Very urgent	329 (12.9)	204 (13.1)	
Urgent	1285 (50.5)	804 (51.6)	
Standard	605 (23.8)	318 (20.4)	
See and treat	89 (3.5)	110 (7.1)	
Redirect or medical expected	18 (0.7)	13 (0.8)	
**Referred**	**SHHP, *n* =** **2545**	**Other, *n* =** **1554**	*P* = 0.1
No	1729 (67.9)	1111 (71.3)	
Yes	816 (32.1)	448 (28.7)	

^a^ *N* = 1124 patients; *n* = 101 excluded because of registration with a non-local GP or with no GP. SHHP = specialist homeless health provider.

Reasons for attendance were broadly similar between groups. SHHP patients were more likely to attend for drug and alcohol-related issues and less likely to attend for mental health reasons (Table 1).

There was no significant difference in triage category between groups. Most attendances (70%) from both groups were in the immediate resuscitation, urgent, or very urgent triage categories. There was no significant difference in referral rates between groups with approximately 30% from both groups being referred for a specialist opinion or admission in the A&E department.

### Frequency of attendance

Repeat attendance was common, with 18% of the total sample attending ≥5 times during the study period. Frequency of attendance was calculated for each patient and compared between the SHHP patients and non-SHHP patients. There was no statistically significant difference between the two groups in the frequency of patients with attendances of 1–4 or ≥5 attendances in the study period (Table 2). The mean attendance over the 3-year study period did not differ between the two groups.(Table 3)

**Table 2.** Frequency of attendance, *N* = 1124

**Frequency of attendance during study period**	**SHHP count (% of SHHP patients**), *n* = 670	**Other count (% of other patients**), *n* = 454	**Significance**
1	276 (41.2)	214 (47.1)	*P* = 0.24
2	137 (20.4)	85 (18.7)	
3	69 (10.3)	53 (11.7)	
4	49 (7.3)	27 (5.9)	
≥5	139 (20.7)	75 (16.6)	

SHHP = specialist homeless health provider.

**Table 3.** Mean attendances

**Mean attendances over 2.5** **years**	*n*	**Standard deviation**	
SHHP	3.8	6.0	*P* = 0.08
Other	3.6	7.6	
**Mean repeat attendances (excluding one-time attenders** **)**		
SHHP	5.8	7.2	*P* = 0.251
Other	5.9	9.5	

SHHP = specialist homeless health provider.

### Effect of age and sex

The frequency of attendance by age group and sex was also analysed to examine if either sex or age was predictive of more frequent attendance. In this sample there was no statistically significant variation in attendance between the SHHP and other GP practice groups by age group or sex (Tables 4 and 5).

**Table 4.** Attendance by sex

**Attendances during study period^a^**	1	2	3	4	≥5
Male, *n* (%)	335 (41.9)	164 (20.45)	90 (11.3)	53 (6.6)	158 (19.8)
Female, *n* (%)	155 (47.8)	58 (17.9)	32 (9.9)	23 (7.1)	56 (17.3)
Significance, Mann-Whitney U test	*P* = 0.108				

^a^Percentages calculated from relevant sex *N*-value; male *n* = 800; female *n* = 324.

**Table 5.** Attendance by age range (years)

Attendances over study period, *n* (%)^a^	1	2	3	4	≥5
18–24	65 (43.0)	32 (21.2)	21 (13.9)	5 (3.3)	28 (18.5)
25–29	66 (38.2)	32 (18.5)	23 (13.3)	17 (9.8)	35 (20.2)
30–34	93 (42.1)	35 (15.8)	28 (12.7)	17 (7.7)	48 (21.7)
35–39	89 (46.6)	44 (23.0)	14 (7.3)	14 (7.3)	30 (15.7)
40–44	61 (40.7)	35 (23.3)	16 (10.7)	11 (7.3)	27 (18.0)
45–49	46 (50.5)	20 (22.0)	5 (5.5)	3 (3.3)	17 (18.7)
50–54	27 (44.3)	14 (23.0)	6 (9.8)	4 (6.6)	10 (16.4)
55–59	23 (50.0)	5 (10.9)	7 (15.2)	2 (4.3)	9 (19.6)
60–64	12 (52.2)	1 (4.3)	0 (0.0)	2 (8.7)	8 (34.8)
65–69	3 (33.0)	3 (33.0)	1 (11.0)	0 (0.0)	2 (22.2)
≥70	5 (62.0)	1 (12.5)	1 (12.5)	1 (12.5)	0 (0.0)
Significance, Kruskal-Wallis	*P* = 0.5				

^a^Percentages calculated from relevant age group *N*-value.

## Discussion

### Summary

This study compared A&E attendances in people experiencing homelessness between those registered with a mainstream GP and those registered with a SHHP over a 2.5-year period. There was no significant difference between attendance rates, with both groups having a high rate of repeat attendance to A&E. Most attendances were urgent or very urgent. The patients registered with the SHHP were older, more likely to be male, and were more likely to attend for drug and alcohol-related reasons. Those registered with a mainstream GP were younger and more likely to attend for mental health-related reasons. Most patients experiencing homelessness identified in the study had a registered local GP.

### Strengths and limitations

The homeless population in any geographical area is difficult to determine as it is inherently fluid, with people moving in and out of homelessness within the area and changing geographical locations frequently. A strength of this study was using several measures to assess whether someone was experiencing homelessness or not and using a broad definition of homelessness that ensured people in temporary accommodation were included in the final analysis. Despite this, it is likely that some people experiencing homelessness were excluded, but this method of determining the homeless population gave the most complete data available.

A further strength was the completeness of the A&E data, which included all attendances, and reasons for attendance were also captured. Limitations included the lack of background medical information about the patient's medical history, which meant it was not possible to assess differences between the two groups of patients besides age and sex.

### Comparison with existing literature

To the authors’ knowledge, this is the first study to examine whether type of primary care provided to people experiencing homelessness altered the rate of attendance to A&E departments. High rates of repeat attendance to A&E were found (mean attendance of 3.7 over 2.5 years in both groups in comparison with a population average of <1 in 5 years^17^). Other studies have also examined patients experiencing homelessness attendances to A&E.^18^ The most recent large study of homeless A&E attendance in Ireland had higher rates of A&E attendance (20 times the population average) and similar demographics of patients than the present study, with those attending most frequently being males aged between 30 and 45 years.^7^ In comparison with the Irish study, patients in the present study attended more frequently for drug and alcohol-related reasons, especially in the SHHP group of whom 24.3% attendances were for drug and alcohol-related reasons as opposed to 14.2% of people in homelessness attendances in the Irish data.^7^

In another study of repeat attenders to A&E, being older and male sex have been found to be predictive of frequent attendance;^19^ however, there was no evidence age or sex had an effect on attendance rates in this group. Although males made up a larger proportion of the group overall, the attendance rates were similar between different ages and groups.

A further study of homeless attenders to the A&E department in London suggested that people experiencing homelessness were using A&E as a substitute for primary care, as 75% of homeless attenders did not know or did not have a registered GP.^20^ In 93% of attendances in the current study the patient had a registered local GP. While access to GP appointments may be difficult, the SHHP provides daily walk-in clinics that any patient can attend, so if accessing appointments was a difficulty it would have been expected there would be a difference in attendance rates.

### Implications for research and practice

This study confirms previous findings that people experiencing homelessness frequently use unscheduled care, but found this was unrelated to the type of primary care they had access to.

Although it has been estimated up to 40% of all A&E admissions in the general population are avoidable,^21^ there is limited evidence that any particular measures make a difference to A&E attendance in the general population;^22^ therefore, it is perhaps not surprising that one particular type of primary care did not reduce A&E attendance.

Without looking in detail at the reasons for attendance, it is not possible to say whether attendances were preventable or not; however, this group of patients is known to have high-health needs, which is reflected in their regular attendance. The Scottish homelessness study^14^ showed a peak in health activity around the time of becoming homeless and that for people in repeated homelessness they had ongoing high-health activity. In this present study, there was no way of distinguishing whether a patient was repeatedly experiencing homelessness or if their homelessness was short-term only. Future studies could look at the smaller numbers of patients with very high levels of attendance as these patients are where interventions are likely to be most beneficial in terms of improving their health.

This study looked only at A&E attendance rates; therefore, it should not be taken from this that there are no benefits to providing specialist primary care for people experiencing homelessness. Services such as the SHHP may have a multitude of benefits, including providing continuity of care, providing preventive health care tailored to the needs of a high-risk demographic, and a multidisciplinary approach that can help with the root causes of patients' homelessness and poor health. This sort of service is not related to A&E attendance and would be difficult to provide in a mainstream primary care service.

Although the SHHP provides a holistic service with drug, alcohol, and mental health support, there is no out-of-hours primary care cover specifically for people experiencing homelessness (out-of-hours primary care in Scotland is provided through a centralised service). Any street outreach or out-of-hours contact is on a voluntary basis by staff. It is possible that a specialised service offering more extended hours or evening outreach might have an impact on emergency department attendance and, therefore, improve health outcomes for vulnerable patients. In Ireland patients attending a mobile health clinic had said they would have attended A&E had the service not been available;^23^ therefore, perhaps street outreach or mobile units could reduce A&E use in this vulnerable group.
